# Syntactic Comprehension in Patients with Amyotrophic Lateral Sclerosis

**DOI:** 10.1155/2014/230578

**Published:** 2014-04-06

**Authors:** Kentarou Yoshizawa, Nao Yasuda, Michinari Fukuda, Yumi Yukimoto, Mieko Ogino, Wakana Hata, Ikuyo Ishizaka, Mari Higashikawa

**Affiliations:** ^1^Department of Rehabilitation, Kitasato University East Hospital, 2-1-1 Asamizodai, Minami-ku, Sagamihara, Kanagawa 252-0380, Japan; ^2^School of Allied Health Sciences, Kitasato University, 1-15-1 Kitasato, Minami-ku, Sagamihara, Kanagawa 252-0374, Japan; ^3^Department of Neurology, School of Medicine, Kitasato University, 1-15-1 Kitasato, Minami-ku, Sagamihara, Kanagawa 252-0374, Japan

## Abstract

Recent neuropsychological studies of patients with amyotrophic lateral sclerosis (ALS) have demonstrated that some patients have aphasic symptoms, including impaired syntactic comprehension. However, it is not known if syntactic comprehension disorder is related to executive and visuospatial dysfunction. In this study, we evaluated syntactic comprehension using the Syntax Test for Aphasia (STA) auditory comprehension task, frontal executive function using the Frontal Assessment Battery (FAB), visuospatial function using Raven's Coloured Progressive Matrices (RCPM), and dementia using the Hasegawa Dementia Scale-Revised (HDS-R) in 25 patients with ALS. Of the 25 patients, 18 (72%) had syntactic comprehension disorder (STA score < IV), nine (36%) had frontal executive dysfunction (FAB score < 14), six (24%) had visuospatial dysfunction (RCPM score < 24), and none had dementia (HDS-R score < 20). Nine of the 18 patients with syntactic comprehension disorder (50%) passed the FAB and RCPM. Although sample size was small, these patients had a low STA score but normal FAB and RCPM score. All patients with bulbar onset ALS had syntactic comprehension disorder. These results indicate that it might be necessary to assess syntactic comprehension in patients with bulbar onset ALS. The implications of these findings are discussed in relation to the pathological continuum of ALS.

## 1. Introduction

Amyotrophic lateral sclerosis (ALS) has historically been considered a neurodegenerative disease characterized by the progressive involvement of upper and lower motor neurons at the bulbar and spinal level. However, the consensus criteria have changed, and ALS is now considered a multisystem disorder in which motor system deficits are prominent but nonmotor deficits can also be observed [1]. Phukan et al. [2] reported that, of 160 patients with ALS, 14% fulfilled the Neary criteria for frontotemporal dementia, 21% had executive dysfunction without dementia, and 14% had cognitive impairment without dementia or executive dysfunction.

The frontal lobe contributes to executive function, language function, and elementary motor function. Executive function refers to higher-level cognitive functions that contribute to the control and direction of lower-level functions such as language, cognition, behavior, and memory [3]. There have been many reports of executive function in patients with ALS, and they have consistently shown that fluency, set-shifting, attention, inhibition, and working memory are impaired [4–6].

Patients with motor neuron disease (MND) and ALS also exhibit language dysfunction, including aphasic symptoms, such as Broca's aphasia, due to frontal lobe deterioration [7–11]. A Japanese account written by Watanabe described paragraphia of an aphasic nature in bulbar onset ALS [12, 13]. In Japan, there were some other reports about writing errors in patients with ALS [14–17]. Cobble [18] assessed nine MND patients on a range of standardized language assessments and found deficits on tasks involving naming, auditory comprehension of complex sentences, semantics, and spelling. In particular, there was a highly significant difference in the auditory comprehension of complex sentences between MND patients and healthy control subjects. Indeed, there are several reports of syntactic comprehension disorder in patients with MND [19–21]. Bak et al. [20] reported that five of six patients with MND had impaired syntactic comprehension, and comprehension of verbs was consistently more impaired than that of nouns. Postmortem examination confirmed the clinical diagnosis of MND-dementia in three of these patients, and the verb disadvantage was associated with prominent pathological changes in Brodmann areas 44 and 45 (Broca's area). It is known that the inferior frontal gyrus (Broca's area) is important for syntactic comprehension [22, 23], and it has been suggested that a neural network including the parietal-temporal region may also be important [24, 25].

Bak et al. [20] reported that visuospatial skills, tested through copying drawings and the visual object and space perception battery, were relatively well preserved in MND, and Neary et al. [26] reported that spatial disorder was absent in three of four MND patients, including two with advanced disease.

Despite these reports, it remains unclear how syntactic comprehension disorder is related to executive and visuospatial dysfunction in ALS. The purpose of this study was to investigate the prevalence and profile of syntactic comprehension in ALS and to investigate the relation of syntactic comprehension with executive and visuospatial function. In addition, single-photon emission computed tomography (SPECT) was performed for two patients to investigate the pathological continuum of ALS.

## 2. Methods

### 2.1. Participants

**Figure 1:**
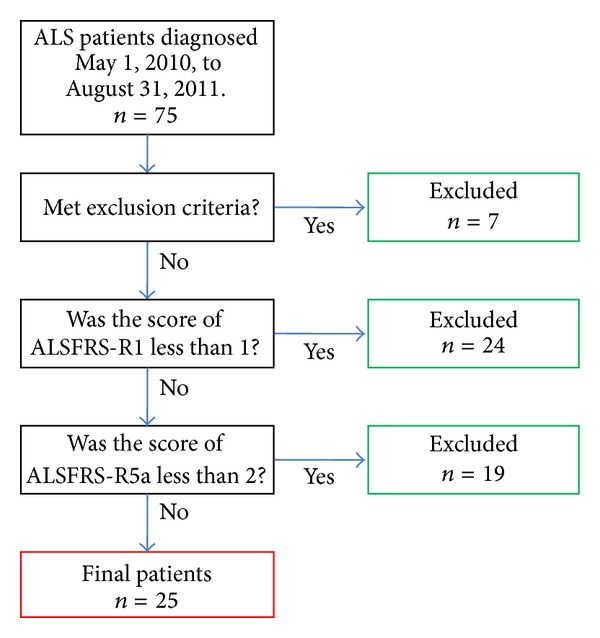
Flowchart showing the sequence of participant selection.

Seventy-five Japanese ALS patients visited the Department of Speech Therapy at Kitasato University East Hospital from May 1, 2010, to August 31, 2011. All fulfilled the El Escorial criteria for definite ALS [27]. Exclusion criteria included past history of neurological, psychiatric, or mental disorder, including schizophrenic disorder and manic-depressive psychosis. Patients were also excluded if they had dysarthria or upper limb impairments that were severe enough to prevent completion of the neuropsychological assessments. There were only 25 patients from the total of 75 who met the inclusion criteria (Figure 1). Patients gave informed consent according to the Helsinki Declaration and the Ethics Committee at Kitasato University School of Medicine approved this study.

### 2.2. Patient Characteristics

Patients were classified with bulbar, upper limb, or lower limb onset ALS by a neurologist according to self-reported initial symptoms. Subscales of the Japanese version of the revised ALS functional rating scale (ALSFRS-R) [28] were used to estimate the severity of dysarthria (ALSFRS-R1, speech subscale) and upper limb impairments (ALSFRS-R5a, cutting food and handling subscale).

### 2.3. Neuropsychological Assessment

We evaluated syntactic comprehension, frontal executive function, visuospatial function, and dementia in all patients. Each of the tests used is characterized by brevity and was selected to minimize the burden on participants. 

*Syntactic Comprehension.* There is a hierarchy of syntactic comprehension in patients with aphasia, not only in English-speaking countries but also in Japan [29–31]. We evaluated syntactic comprehension using the Test of Syntactic Processing in Aphasia (Syntax Test for Aphasia; STA) [32], which was designed to assess syntactic aspects of language in patients with aphasia in Japan. The STA consists of an auditory comprehension task, a reading comprehension task, and a sentence production task. We used only the auditory comprehension task, which is designed to assess comprehension of active and passive sentences with regular and nonregular word order and to determine the use of word meaning, word order, or particles.

The STA auditory comprehension task includes four levels, with eight sentences in each level. Level I consists of nonreversible, active sentences with regular word order; Level II consists of reversible, active sentences with regular word order; Level III consists of reversible, active sentences with regular and nonregular word order; and Level IV consists of reversible, passive sentences with regular and nonregular word order (Table 1).

For each sentence, patients are presented with four to six pictures and are required to point to the picture that corresponds to the sentence read by the examiner. Seven sentences within a level have to be answered correctly to pass that level. Failure to pass all four levels (STA score < IV) was classed as failure of the STA auditory comprehension task and was considered indicative of syntactic comprehension disorder.

In Japanese grammar, particles are short words that follow the modified noun, verb, or adjective and can indicate various functions and meanings within a sentence. Some particles are equivalent to English prepositions, but others have a unique usage that is not found in English. For example, the sentences in Level III (Table 1) show that substitution of particles such as “ga” and “wo” makes reversible meaning.

*Frontal Executive Function.* The Frontal Assessment Battery (FAB) is a short battery of tests that assess frontal executive function [33]. It has six subtests: conceptualization, mental flexibility, motor programming, sensitivity to interference, inhibitory control, and environmental autonomy. Terada et al. reported that the mean ± standard deviation total score for normal healthy adults (mean age, 64.4 ± 8.3 years) was 14.7 ± 1.3 [34]. Therefore, a score less than 14 out of 18 was classed as failure of the FAB test and was considered indicative of frontal executive dysfunction.

*Visuospatial Function.* Raven's Coloured Progressive Matrices (RCPM) are a standardized tool for both geriatric and pediatric populations [35] and were used to assess visuospatial function. In RCPM, patients are required to select one picture out of six that is the same in pattern. We used three picture sets (A, AB, and B) that each included 12 pictures, for a total of 36 pictures. A score of less than 24 out of 36 was classed as failure of the RCPM test and was considered indicative of visuospatial dysfunction. The RCPM requires the ability to analyze color, form, and linear slope. These visual processing tasks take place in different subdivisions of the visual association areas (primarily the occipital lobe) [36]. 

*Dementia.* The Hasegawa Dementia Scale-Revised (HDS-R) is a screening test for patients with dementia in Japan that is similar to Mini-Mental State Examination and correlates well with Mini-Mental State Examination [37]. A score of less than 20 out of 30 was classed as failure of the HDS-R and considered indicative of dementia.

### 2.4. Neuroimaging

In two patients (patients 7 and 22) who had consented to go through neuroimaging test in writing, we performed ^123^I-isopropyl amphetamine SPECT (IMP-SPECT). Both patients selected for IMP-SPECT had similar clinical characteristics: they were both women aged between 70 and 80 years with upper limb onset ALS. The disease duration was between 1 and 2 years. Regional cerebral blood flow (r-CBF) was assessed using three-dimensional stereotactic surface projection.

### 2.5. Statistical Analysis

Relations between syntactic comprehension (STA auditory comprehension score), frontal executive function (FAB score), visuospatial function (RCPM score) and demographic variables (age, disease duration, severity of dysarthria (ALSFRS-R1 score), and severity of upper limb impairment (ALSFRS-R5a score) were assessed using Pearson's correlation. Correlations among the neuropsychological tests (STA auditory comprehension, FAB, RCPM, and HDS-R) were calculated with Spearman rank correlation coefficient. All analyses were performed using SPSS version 10.0 J software for Windows. Data are presented as mean ± standard deviation unless otherwise stated.

## 3. Results

### 3.1. Patient Characteristics

Results are presented from 25 patients (16 men, 9 women) aged 67.9 ± 9.0 years. Disease duration was 23.9 ± 15.5 months (range, 6–61 months). The ALSFRS-R1 score was 3.5 ± 0.8 and the ALSFRS-R5a score was 3.6 ± 0.5. Eleven patients were classified with bulbar onset ALS, 10 with upper limb onset ALS, and four with lower limb onset ALS. The clinical and neuropsychological characteristics of patients are summarized in Table 2.

### 3.2. Neuropsychological Assessment

Eighteen out of the 25 patients (72%) failed to complete all four levels of the STA auditory comprehension task (score < IV; Table 2) and were classed as having syntactic comprehension disorder. Of the 18 patients, 12 (66.8%) failed to comprehend reversible, passive sentences with regular and nonregular word order and were classed at level III. Three (16.6%) of the 18 patients failed to comprehend reversible, active sentences with regular and nonregular word order and were classed at level II. Other 3 (16.6%) of the 18 patients failed to comprehend reversible, active sentences with regular word order, and were classed at level I. There were more errors for reversible sentences than for nonreversible sentences, and more errors for passive sentences than for active sentences. The FAB score was 14.4 ± 3.5 (range, 4–18). Nine out of the 25 patients (36%) failed the FAB (score < 14; Table 2) and were classed as having frontal executive dysfunction. The RCPM score was 28.2 ± 5.4 (range, 14–36). Six out of the 25 patients (24%) failed the RCPM (score < 24; Table 2) and were classed as having visuospatial dysfunction. Mean HDS-R score was 27.3 ± 3.0, which is within normal limits. No patient scored less than 20 on the HDS-R; therefore, no patients were classed as having dementia (Table 2). Of the 18 patients who failed the STA auditory comprehension task, nine (50%) also failed the FAB and six (33%) failed both the FAB and the RCPM.

Patients were divided into four groups (Table 3). Group A passed all four tests (*n* = 7); Group B failed the STA auditory comprehension task but passed the FAB, the RCPM, and the HDS-R (*n* = 9); Group C failed the STA auditory comprehension task and the FAB but passed the RCPM and the HDS-R (*n* = 3); and Group D failed the STA, the FAB, and the RCPM (*n* = 6). Of the seven patients in Group A, four had upper limb onset ALS and three had lower limb onset ALS. Of the nine patients in Group B, one had upper limb onset ALS, two had lower limb onset ALS, and six had bulbar onset ALS. All three patients in Group C had bulbar onset ALS. Of the six patients in Group D, four had upper limb onset ALS and two had bulbar onset ALS. All patients with bulbar onset ALS failed the STA auditory comprehension task (Table 3).

The scores on the STA auditory comprehension task, the FAB, and the RCPM were not correlated with age, disease duration, ALSFRS-R1 score, or ALSFRS-R5a score (Table 4). The STA auditory comprehension score had strong positive correlation with the FAB score (rs = 0.791, *P* < 0.05) but moderate positive correlation with the RCPM score (rs = 0.600, *P* < 0.05), and the FAB score also showed strong positive correlation with the RCPM score (rs = 0.734, *P* < 0.05) (Table 5).

### 3.3. Neuroimaging

**Figure 2:**
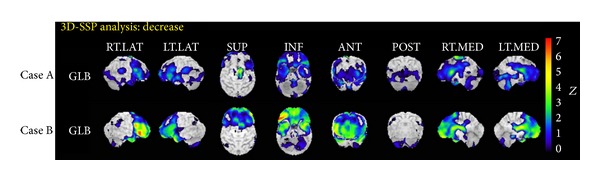
3D-SSP Analysis. Case A. IMP-SPECT images revealed mildly reduced r-CBF in the bilateral frontal lobes of patient 7, who was within normal limits at all of three tests. Case B. IMP-SPECT images revealed moderately reduced r-CBF in the bilateral frontotemporal lobes of patient 22, who failed all three tests.

Patient 7 passed all four levels of the STA auditory comprehension task and passed the FAB, the RCPM, and the HDS-R (Group A). IMP-SPECT revealed that she had mildly reduced r-CBF in the bilateral frontal lobes. Patient 22 passed the HDS-R but failed the STA auditory comprehension task, the FAB, and the RCPM (Group D) and had moderately reduced r-CBF in the bilateral frontotemporal lobes (Figure 2).

## 4. Discussion

The majority (72.0%) of ALS patients tested in this study had syntactic comprehension disorder, which is one of the linguistic characteristics of ALS with aphasic symptoms [18–21]. However, we decided to exclude the patients with severe dysarthria. We should take into account that we might have underestimated the frequency of syntactic comprehension disorder, because the language disorders are often associated with the bulbar presentation. The prevalence of syntactic comprehension disorder in MND or ALS patients varies across the literature, ranging from 27.8% to 83.3% (Table 6). Rakowicz and Hodges [19] reported that four out of 15 patients with bulbar onset MND had syntactic comprehension disorder. Cobble [18] reported a single patient with bulbar onset MND who had syntactic comprehension disorder, and Bak et al. [20] reported that five out of six patients with bulbar onset MND had syntactic comprehension disorder. In our cohort, all patients with bulbar onset ALS had syntactic comprehension disorder, and it is therefore possible that bulbar onset ALS is associated with syntactic comprehension disorder.

The syntax test used in this study evaluated the strategy level (use of word meaning, word order, or particle) of syntactic comprehension [38]. Patients with syntactic comprehension disorder were more impaired at the level of use of particle (Level III) than use of word meaning (Level I) and word order (Level II). The errors depended on the complexity of syntax. This pattern of errors is similar to that observed in aphasic patients [39, 40]. ALS patients progressively develop disorders of verbal and literal expression due to dysarthria or upper limb weakness, and syntactic comprehension disorder might therefore be concealed when not specifically tested for in clinical settings.

Bak et al. [20] reported that visuospatial skills were relatively well preserved in MND patients who had impaired syntactic comprehension. Moreover, Phukan et al. [2] reported that of 19 ALS patients who had multidomain executive impairment, only nine had visuospatial impairment. In our cohort, all patients who failed the RCPM or the FAB also failed the STA auditory comprehension task. Bak et al. [20, 41, 42] reported that MND with aphasic symptoms and MND with dementia were extremes on a nosological continuum with a varying degree of overlap between them. According to this theory, our data (Table 5) might indicate that ALS also represents a continuum including aphasia, executive dysfunction, and visuospatial dysfunction. However, the present study included some ALS patients with aphasic symptoms who did not show executive dysfunction or visuospatial dysfunction.

Neuropsychological and neuroimaging studies have reported that Broca's area (left inferior frontal gyrus, Brodmann's areas 44 and 45) is involved in the processing of sentence structure [43–46]. We found a moderate reduction in r-CBF in the bilateral frontal lobes of a patient who failed the STA auditory comprehension task, the FAB, and the RCPM but only a mild reduction in a patient who passed these tests. This may indicate that neurodegeneration in the bilateral frontal lobes underlies the results of our neuropsychological evaluations. However, there is no evidence of a relation between neuroimaging data and syntactic comprehension in present study, and further research of r-CBF in ALS patients is needed.

Broca's area is adjacent to the lower precentral gyrus, which is the motor center for the face, lips, tongue, and pharynx. Therefore, we propose that, in patients with bulbar onset ALS, neurodegeneration may have progressed from the medulla oblongata and pons to the precentral gyrus and inferior frontal gyrus. Six of 10 (60.0%) patients with upper limb onset ALS had syntactic comprehension disorder, and 1 of 4 (25.0%) patients with lower limb onset ALS had syntactic comprehension disorder (Table 3). If neurodegeneration first progressed from the medulla oblongata and pons to the upper prefrontal gyrus and Broca's area, then neurodegeneration might have occurred in some patients with upper or lower limb onset type ALS.

Taylor et al. [47] found language domain impairment in 43% of patients with ALS, and executive domain impairment in 31%. They concluded that although the two domains were strongly associated, executive dysfunction did not fully account for the language impairment observed. These were similar to our results. In our cohort, there was strong correlation between the score of the STA auditory comprehension task and the FAB. On the other hand, there were 9 patients who failed level IV on the STA auditory comprehension task but passed the FAB and RCPM (Group B). This may indicate that neurodegeneration was limited to Broca's area and had not progressed to the prefrontal lobe. Furthermore there were 3 patients who failed the STA auditory comprehension task and the FAB but passed the RCPM (Group C). In addition, there were no patients who failed the FAB and/or the RCPM but passed the STA auditory comprehension task and who failed the RCPM but passed the FAB. These results raise the possibility that neurodegeneration in ALS may sequentially progress from Broca's area to the prefrontal lobe and occipital lobe. Longitudinal studies on the relation between neuropsychological evaluations and neuroimaging data, including IMP-SPECT, in patients with ALS are required. These will help to elucidate the mechanisms by which neurodegeneration progresses in ALS.

Ichikawa et al. [14] reported that, of 15 patients with bulbar onset type ALS who had writing errors, six showed grammatical errors. However, evaluation of the abilities of syntactic comprehension and writing at the same time has not yet been performed. Further studies are needed in order to confirm the relation between writing errors and syntactic comprehension disorder.

## 5. Conclusion

In this study we investigated the prevalence and profile of syntactic comprehension in ALS and the relation of syntactic comprehension with executive and visuospatial function. There was a high prevalence of syntactic comprehension disorder, especially in patents with bulbar onset ALS. These results raise the possibility that neurodegeneration in ALS may sequentially progress from Broca's area to the prefrontal lobe and occipital lobe.

## Figures and Tables

**Table 1:** Each stage and examples of Syntax Test for Aphasia.

Level	Strategy	Definition	Sample of Japanese (English)
I	The meaning of a word	Nonreversible, active sentences in regular word order	otokonoko (n) ga (p) aruiteiru (v) (The boy is walking.) okasan (n) ga (p) teburu (n) wo (p) fuiteiru (v) (The mother is wiping the table.)

II	The word order	Reversible, active sentences in regular word order	onnanoko (n) ga (p) otosan (n) ni (p) purezento (n) wo (p) ageteiru (v) (The girl is giving a present to the father.) okasan (n) ga (p) otokonoko (n) wo (p) ositeiru (v) (The mother is pushing the boy.)

III	The particle without complementizer	Reversible, active sentences in regular and nonregular word order	otousan (n) wo (p) onnanoko (n) ga (p) ositeiru (v) (The father is pushed by the girl.) otousan (n) ga (p) onnanoko (n) wo (p) ositeiru (v) (The father pushed the girl.)

IV	The particle with complementizer	Reversible, passive sentences in regular and nonregular word order	otosan (n) ga (p) onnanoko (n) ni (p) rinngo (n) wo (p) moratteiru (v) (The father is given an apple from the girl.) otosan (n) ni (p) onnanoko (n) ga (p) rinngo (n) wo (p) moratteiru (v) (The girl is given an apple from the father.)

(n): noun; (v): verb; (p): particle.

**Table 2:** Patient characteristics and the results of neuropsychological assessments.

Patient number	Age (years)	Gender	handedness	Subtype	Disease duration (months)	ALS FRS-R1 score	ALS FRS-R5a score	HDS-R score	STA achieved highest stage	FAB score	RCPM score
1	53	F	R	Spinal (u)	41	3	4	30	IV	18	30
2	79	F	R	Spinal (l)	6	3	3	29	IV	16	27
3	61	M	R	Spinal (u)	24	4	3	30	IV	17	35
4	59	M	R	Spinal (l)	59	4	3	30	IV	18	32
5	48	M	R	Spinal (u)	32	4	3	30	IV	17	32
6	50	M	R	Spinal (l)	9	4	4	30	IV	18	33
7	74	F	R	Spinal (u)	21	3	3	29	IV	16	27
8	73	M	R	Spinal (u)	24	4	3	29	III	18	30
9	74	M	R	Bulbar	18	4	4	22	III	16	30
10	74	M	R	Spinal (l)	12	4	4	28	III	16	34
11	74	M	R	Bulbar	10	4	4	28	III	17	34
12	68	M	R	Bulbar	14	4	3	28	III	17	30
13	59	M	R	Spinal (u)	33	4	3	30	III	16	36
14	71	M	R	Bulbar	9	4	4	30	III	17	29
15	71	M	R	Bulbar	61	2	4	27	III	16	34
16	73	F	L	Bulbar	20	2	4	29	III	14	31
17	75	F	R	Bulbar	26	3	4	29	II	11	25
18	63	F	R	Bulbar	46	4	3	23	III	13	26
19	72	M	R	Bulbar	10	2	4	30	II	11	25
20	68	F	R	Spinal (u)	10	4	4	25	III	13	24
21	59	F	R	Spinal (u)	11	4	4	25	II	12	18
22	75	F	R	Spinal (u)	13	4	3	26	I	11	24
23	82	M	R	Bulbar	41	3	4	22	III	12	24
24	73	M	R	Bulbar	28	2	4	23	I	8	23
25	69	M	R	Spinal (u)	20	4	4	21	I	5	14

Average	67.9				23.9	3.5	3.6	27.3		14.4	28.2
SD	9.0				15.5	0.8	0.5	3.0		3.5	5.4

ALS FRS-R: revised amyotrophic lateral sclerosis functional rating scale; HDS-R: Hasegawa Dementia Scale-revised; STA: Syntax Test for Aphasia; FAB: Frontal Assessment Battery; RCPM: Raven's Coloured Progressive Matrices; R: right; L: left; (u): upper limb; (l): lower limb.

**Table 3:** The distribution of patients according to the initial symptoms and the results of STA, FAB, and RCPM.

Group	Initial symptoms		
	Upper limb	Lower limb	Bulbar
A (STA+, FAB+, RCPM+)	4	3	0
B (STA−, FAB+, RCPM+)	2	1	6
C (STA−, FAB−, RCPM+)	0	0	3
D (STA−, FAB−, RCPM−)	4	0	2

STA: Syntax Test for Aphasia; FAB: Frontal Assessment Battery; RCPM: Raven's Coloured Progressive Matrices; +: pass, −: failure.

**Table 4:** Correlation between demographic variables and results of STA, FAB, and RCPM.

	Pearson's correlation
Age	
STA score	−0.390
FAB score	−0.311
RCPM score	−0.258
Disease duration	
STA score	0.199
FAB score	0.123
RCPM score	0.210
ALSFRS-R1 score	
STA score	0.168
FAB score	0.258
RCPM score	0.087
ALSFRS-R5a score	
STA score	−0.332
FAB score	−0.348
RCPM score	−0.251

ALS FRS-R: revised amyotrophic lateral sclerosis functional rating scale; STA: Syntax Test for Aphasia; FAB: Frontal Assessment Battery; RCPM: Raven's Coloured Progressive Matrices.

**Table 5:** Spearman rank correlation coefficient among neuropsychological assessments.

	rs	*P* value
STA score		
FAB score	0.791	<0.05
RCPM score	0.600	<0.05
HDS-R score	0.574	<0.05
FAB score		
RCPM score	0.734	<0.05
HDS-R score	0.637	<0.05
RCPM score		
HDS-R score	0.565	n.s.

STA: Syntax Test for Aphasia; FAB: Frontal Assessment Battery; RCPM: Raven's Coloured Progressive Matrices; HDS-R: Hasegawa Dementia Scale-revised; n.s.: not significant.

**Table 6:** Previous reports of the frequency of syntactic comprehension disorders in amyotrophic lateral sclerosis and motor neuron disease.

Study	Tests	Frequency
Doran et al. (1995) [8]	The test of the reception of grammar (TROG) and the shortened version of the token test	3/5 (60.0%)
Rakowicz and Hodges (1998) [19]	The test of the reception of grammar (TROG)	5/18 (27.8%)
Cobble (1998) [18]	The test of auditory comprehension of sentences (PALPA)	5/9 (55.6%)
Bak et al. (2001) [20]	The test of the reception of grammar (TROG)	5/6 (83.3%)
Taylor et al. (2013) [47]	The test of the reception of grammar (TROG)	18/51 (35.3%)
Current results	Syntax test for aphasia (STA)	18/25 (72.0%)
